# AI Acceptability in Dentistry: Insights from Dental Professionals and Students in the Netherlands: A Pilot Study

**DOI:** 10.1016/j.identj.2025.103933

**Published:** 2025-10-07

**Authors:** Ru-shan Goey, Leslie Elenbaas, Erwin Berkhout, David Anssari Moin, Rianne van der Kleij, Tymour Forouzanfar, Niels H. Chavannes, María Villalobos-Quesada

**Affiliations:** a Department of Oral and Maxillofacial Surgery, Leiden University Medical Center, Leiden, The Netherlands; b Department of Oral Radiology & Digital Dentistry, Academic Centre for Dentistry Amsterdam (ACTA), University of Amsterdam and Vrije Universiteit Amsterdam, Amsterdam, The Netherlands; c Promaton Dental AI, Amsterdam, The Netherlands; d National eHealth Living Lab (NeLL), Public Health and Primary Care Department, Leiden University Medical Centre, Leiden, The Netherlands

**Keywords:** Artificial intelligence, Dentistry, Stomatology, Students, Maxillofacial surgeons, Dentists, The Netherlands, Perceptions, Acceptance

## Abstract

**Introduction and aims:**

There is an increasing worldwide interest and investment in the application of Artificial Intelligence (AI), and the dental field is no exception. AI shows promise in all dental disciplines. However, uptake in clinical practice is slow, and there is limited understanding of users’ acceptability in the Netherlands. This study aims to provide insights into the acceptability of Dutch dental professionals and students regarding AI in the dental field.

**Methods:**

This cross-sectional, observational study was conducted using an anonymous online questionnaire (Qualtrics). Participants included dental students, dentists, and maxillofacial surgeons in the Netherlands. Data collection took place between April and May 2023, and data were analyzed using descriptive statistics.

**Results:**

The survey was completed by 166 participants: 61% were dentists, 34% dental students, and 5% maxillofacial surgeons. Overall, respondents were positive about working with AI, regarding it as an opportunity to, for example, save time and improve care in all dental fields. They did not perceive AI as threatening, but as a useful support tool. The tasks for which AI was regarded most useful were: detection of pathologies in the jaws, evaluation of treatment success, planning jaw surgery, detection of caries, endodontic abnormalities, periodontal abnormalities, and 3D implant planning and positioning. AI education was deemed necessary at dental/medical school, postgraduate levels, and conferences.

**Conclusion and clinical relevance:**

This study contributes to filling the literature gap regarding AI acceptability in the dental field and provides evidence to justify the structural incorporation of AI education. It is necessary to prepare current and future dental professionals to work with AI, and to play an active role in the co-development, implementation, and sustained adoption of this technology. Only such an integrated strategy will allow us to generate AI that will positively transform clinical practice.

## Introduction

Worldwide, there is increasing interest and investment in the application of Artificial Intelligence (AI). The dental field is no exception,^1^^,^^2^ with a dramatic increase in publications over the last decade. We define AI as software designed to accomplish a complex goal by interpreting data, processing, deriving knowledge, and determining the best course of action.^3^ Several studies report numerous applications of AI across various fields of dentistry, including oral and maxillofacial radiology and surgery, orthodontics and dentofacial orthopedics, endodontics, periodontics, and forensic odontology. Research indicates that AI in dentistry can improve diagnostic accuracy, support clinical decision-making, assist treatment planning, and predict treatment outcomes.^4^, ^5^, ^6^ The yet unrealized promise of AI in dentistry is to make dental care personalized, predictive, preventive, and participatory.^7^

Although dentistry in high-resource settings is commonly supported by digital technologies and is considered suited for adopting AI, the use of AI systems in dental practice remains low.^7^^,^^8^ The Council of European Dentists, for example, recognizes AI’s potential for “augmenting capabilities, enhancing efficiency and accuracy, as well as reducing costs.” However, it also recognizes the implementation challenges for AI in dentistry and the absolute need to include dental professionals as early as possible in order to generate “operational” systems. It also warns about generating an extra burden for professionals and increasing costs, and strongly criticizes the lack of comprehensive and specific AI legislation for healthcare, which could clarify questions, such as those related to responsibility and liability. Finally, it highlights the need for training the workforce.^9^ Other challenges for AI adoption include limited data availability and representativeness, which can be due to data protection and privacy issues, or inappropriate technical (e.g., lack of interoperability and standards) and organizational structures.^5^^,^^10^ Other reported challenges include problems with bias, discrimination, and fairness, failure to incorporate ethical principles into the design of AI systems, as well as lack of rigor and robustness.^5^^,^^11^ The consequences of these challenges are visible in AI’s adoption rates in dentistry. In the Netherlands, for example, a 2020 report commissioned by the Dutch Ministry of Health indicated that dentistry was the medical field with the lowest adoption of AI applications.^12^

AI in dentistry is expected to quickly gain ground in all dental disciplines.^13^^,^^14^ One factor that will determine the pace of adoption is the acceptability of AI among users (dentists and patients). Acceptability is related to the perceived usefulness and the alignment of a technology with the context (such as users’ goals and needs).^15^^,^^16^ Understanding acceptability can contribute to aligning AI with users’ needs and inform strategies to boost the adoption of the technology, for example, the development of study curricula and professional training programs. It can additionally help to develop implementation strategies that match the context. In some countries, such as South Korea, India, Pakistan, Turkey, Peru, and Brazil, acceptability studies have already been conducted.^17^, ^18^, ^19^, ^20^, ^21^ However, in general, there is still a limited understanding of users’ acceptability of AI in dentistry.^22^ To our knowledge, there is no acceptability data from the Netherlands that can guide prioritization, development, implementation, and education strategies for AI in dentistry. This study aims to fill that gap by providing insights into the acceptability of Dutch dental professionals (dentists and maxillofacial surgeons) and dental students regarding AI in the dental field. Our hypothesis is that Dutch dental professionals and students find AI acceptable.

## Methods

This is a cross-sectional, observational study that used an anonymous questionnaire administered in Qualtrics (version April 2023) as the data-collection tool. The study was approved by the ethical committee of the Academic Centre for Dentistry Amsterdam (ACTA) (ID:75888). This study did not fall under the Dutch Medical Research Involving Human Subjects Act (WMO) and followed institutional guidelines for good research practice and integrity. Participants consented to participate in the study via the online questionnaire. They received information about the goal of the study, the procedures, and the use of data collected, and could contact the researchers with any questions. Data were collected anonymously and stored securely at the Leiden University Medical Centre. Participation was entirely voluntary, completely anonymous, and without financial incentives.

### Data collection instrument

An interdisciplinary team (1 maxillofacial surgeon, 3 dentists, 2 digital health researchers) designed the questionnaire, considering scientific literature^17^, ^18^, ^19^ (supplemental file 1). All dental health professionals (the maxillofacial surgeon and the dentists) had more than 3 years of clinical experience in academic and private settings. Both digital health researchers have multiple years of research experience in AI, with expertise in implementation sciences, qualitative research, validation, ethics, and law. To ensure comprehensibility and relevance, the questionnaire was externally and independently reviewed by 2 dentists and 1 maxillofacial surgeon. The survey comprised 18–19 mandatory questions, depending on participant characteristics (supplemental file 1). All questions except those on age and years of experience were multiple choice. The survey was only offered in Dutch to match the population’s characteristics. Questions included the following information: basic demographics, awareness, and acceptability of possible applications of AI in dentistry.

### Study population and recruitment

The study population included Dutch-speaking dental students, dentists, and maxillofacial surgeons who were invited to participate in the study between April and May 2023. All registered students at ACTA, approximately 900, were invited via email to answer the online questionnaire. ACTA is the largest dentistry faculty in the Netherlands. Similarly, 500 dentists were randomly selected from the Royal Dutch Dental Association (KNMT) registry and invited via email. The KNMT is the professional association for dentists, oral surgeons and orthodontists in the Netherlands, currently has approximately 12,000 members. Email invitations were sent by the researchers. For dentists, the KNMT carried out the random selection of participants, according to their policy. Maxillofacial surgeons were invited to participate in the study through a newsletter of the Dutch Association of Maxillofacial Surgeons (NVMKA), following the association’s policy. The NVMKA includes approximately 340 surgeons and 60 maxillofacial surgery students. All groups received 1 reminder within a month. No other inclusion or exclusion criteria were applied.

### Analysis

Data were exported from Qualtrics to Microsoft Excel. Data from respondents who did not complete section 3, and therefore provided no subsequent data (early drop-offs), were excluded from the analysis (see supplemental file for the complete questionnaire). Data were analyzed using descriptive statistics.

## Results

### Demographics

**Fig. 1:**
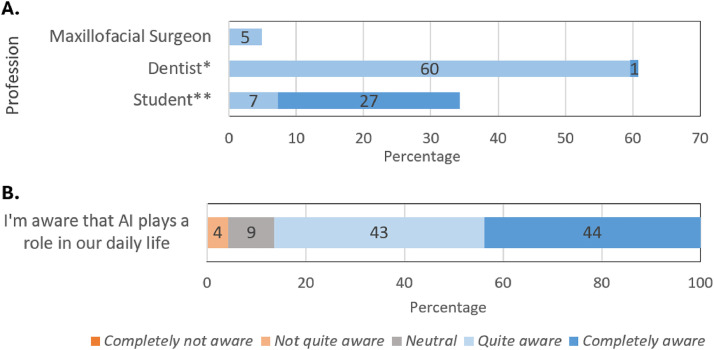
A. Respondents’ dental professions. Data labels indicate percentages. * Light blue indicates dentists, dark blue indicates orthodontists. ** Light blue indicates bachelor’s students; dark blue indicates master’s students. B. Perception of dental students and professionals about the role of AI in their daily lives.

A subset of the surveys (n = 18) was excluded from the analysis due to incomplete data (early drop-offs). The survey was completed by 166 participants: 93 women (56.0%) and 72 men (43.4%); 1 participant (0.6%) did not disclose this information. The mean age of participants was 34 years, ranging from 18 to 69 years. Most participants were dentists (n = 101, 60.8%, mean age 38), followed by dental students (n = 57, 34.3%, mean age 25), and maxillofacial surgeons (n = 8, 4.8%, mean age 39) (Figure 1). Among dentists, 2 were orthodontists (1.2% of the total respondents). Among the students, 12 were bachelor’s students (7.2% of the total respondents) and 45 were master’s students (27.1% of the total respondents).

#### Awareness of AI in daily life

**Fig. 2:**
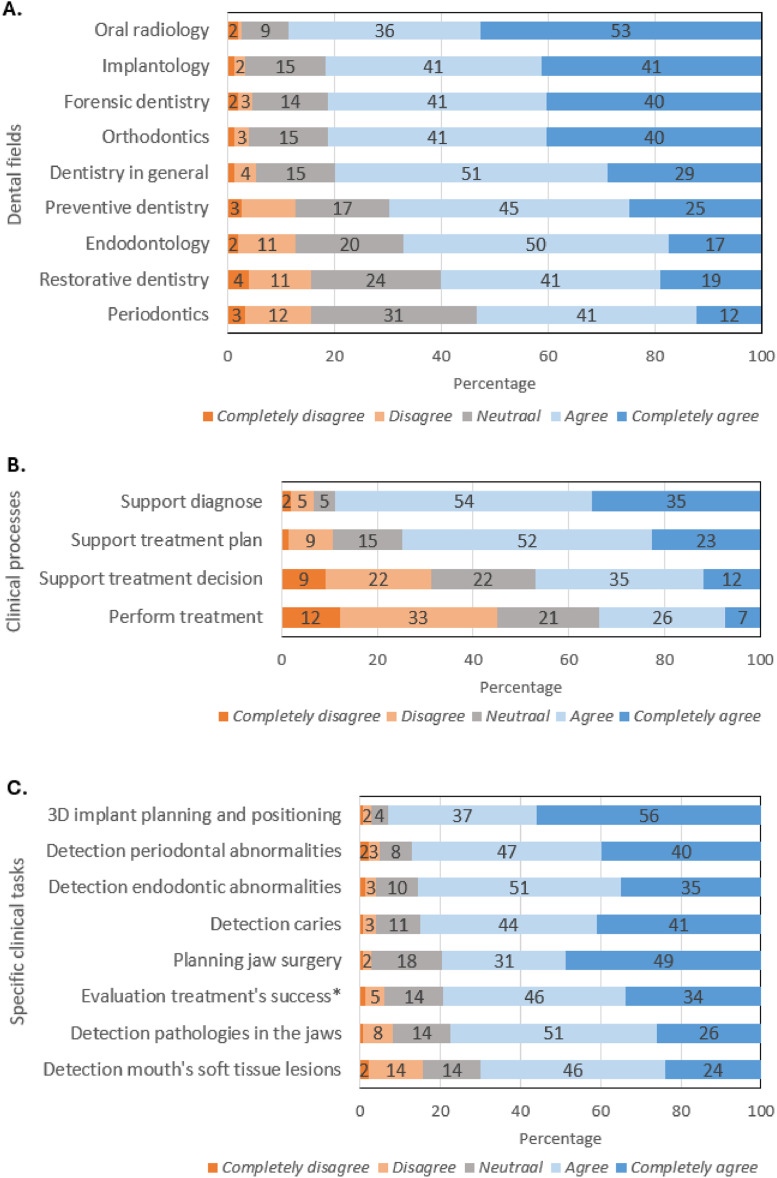
Perceived usefulness of AI in (A) dental fields, (B) general clinical processes, and (C) specific clinical tasks. Percentages ≤ 1% are not displayed as data labels. *As a quality control tool for the evaluation of treatment success.

The survey started by asking respondents about their awareness of AI in daily life; most (n = 140, 86.4%) indicated that they were completely or quite aware of AI (Figure 2).

### Useful applications of artificial intelligence in dentistry

**Fig. 3:**
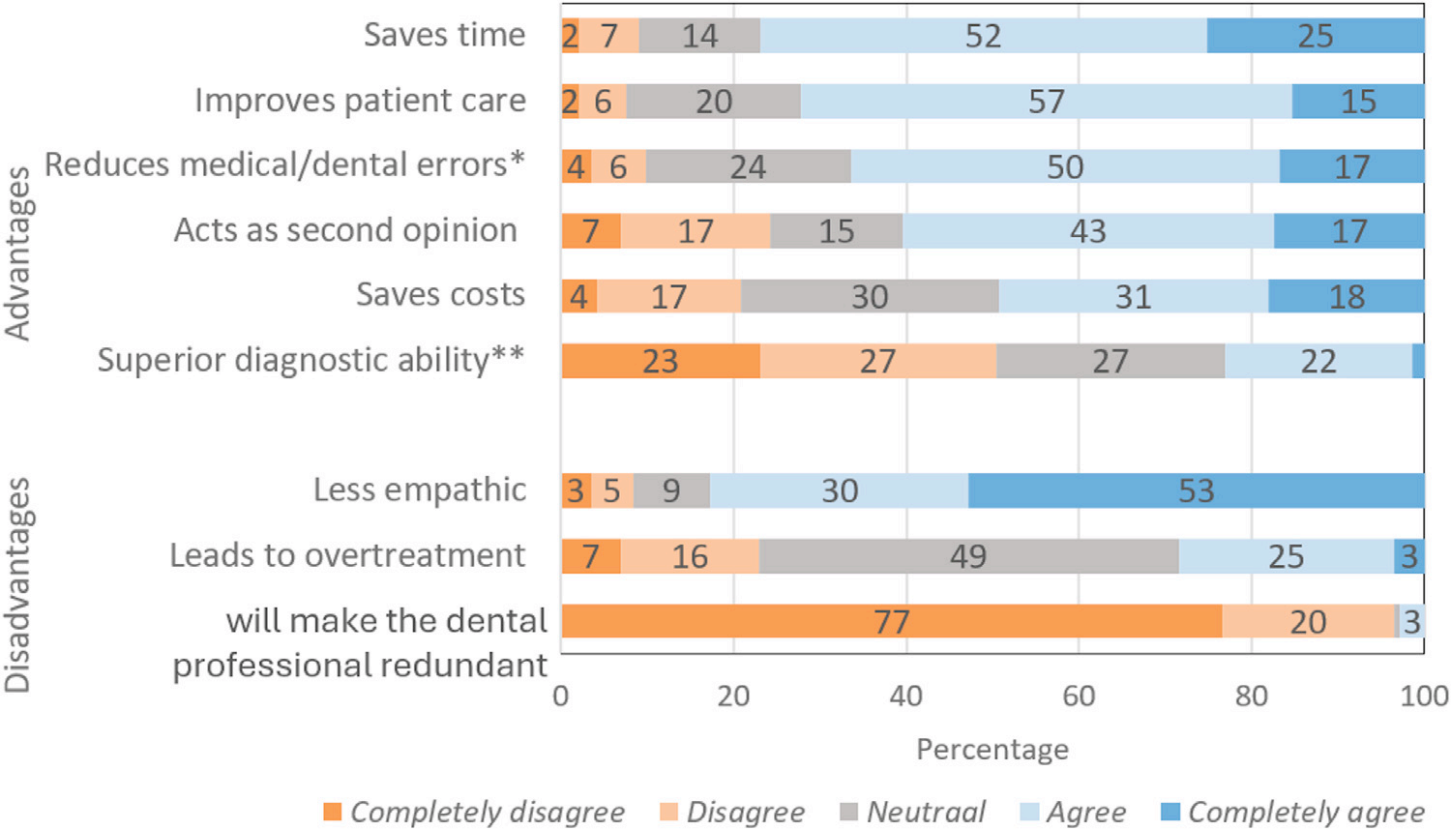
Perception of advantages and disadvantages of AI. Percentages ≤ 1% are not displayed as data labels. *For example, missing a pathology. **In comparison to a dental professional.

Respondents perceived that AI could be useful in nearly all dental fields surveyed (Figure 3A). Between 80-89% of respondents expressed agreement about the usefulness of AI in oral radiology, implantology, orthodontics, forensic dentistry, and dentistry in general. A smaller percentage of respondents (57–70%) expressed agreement that AI is useful in preventive dentistry, endodontology, restorative dentistry, and periodontics. The percentage of respondents who did not deem AI useful in these fields was very low, ranging from 3–15%.

Respondents expressed agreement about AI’s usefulness for supporting clinical processes such as making diagnoses and establishing treatment plans (89% and 75% of respondents, respectively) (Figure 3B). AI that supports general clinical processes, such as treatment decision-making and performing treatments, was perceived as less useful (47% and 33% of respondents expressed agreement).

Respondents were then asked about relevant applications of AI to support clinical tasks (Figure 3C), which were examples of the clinical processes presented in Figure 3B. Most respondents expressed agreement (between 70–93%) on the usefulness of AI in all examples provided. The task most often regarded useful by respondents was 3D implant planning and positioning (93%).

### Advantages and disadvantages of AI in dentistry

**Fig. 4:**
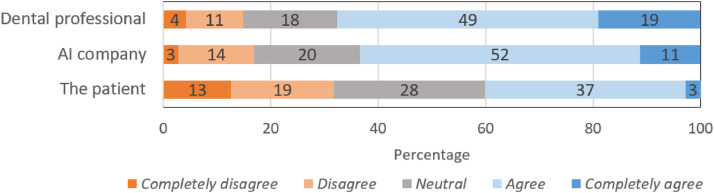
Dental professionals, AI companies, and patients as potential subjects of liability for medical or legal problems caused by AI.

Most respondents expressed agreement regarding the time-saving aspect of AI and the potential to improve patient care (77% and 72% respectively) (Figure 4). A substantial proportion of respondents agreed that AI could reduce medical/dental errors, provide support to professionals as a second opinion, and save costs (49–66% of respondents expressed agreement). Respondents were neutral about AI having a “superior diagnostic ability compared to dental professionals”, with 50% of respondents expressing disagreement and 23% expressing agreement with the statement.

Most respondents (83%) agreed that AI has a “limited empathy in comparison with a dental professional”. Respondents were mainly neutral (49%) about the risk of overtreatment. Finally, respondents strongly expressed disagreement (97%) with the statement that “AI will make dental professionals redundant.”

### Liability caused by AI

**Fig. 5:**
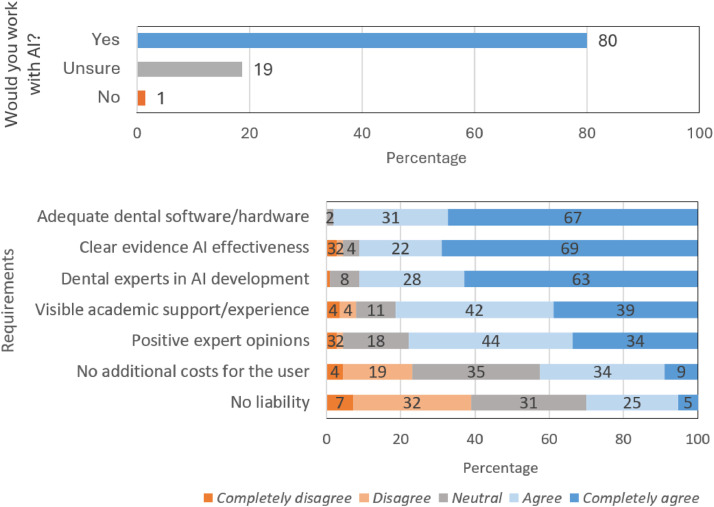
Readiness (A) and requirements to work with AI (B). Percentages ≤1% are not displayed as data labels.

When asked about liability (Figure 5), respondents expressed agreement that dental professionals and AI companies (68% and 63%, respectively) should be held liable for medical or legal problems caused by AI. In contrast, only 40% expressed agreement that patients should be held liable.

### Using AI in daily practice

**Fig. 6:**
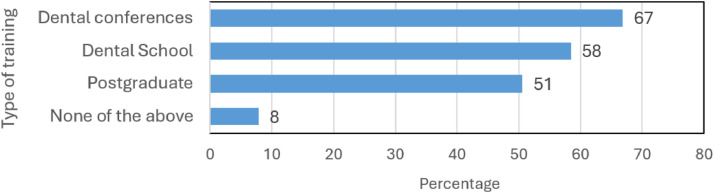
Integration of AI education into the training of dental students and professionals (multiple responses allowed).

The majority (80%) of respondents indicated that they would use AI in their role as dental professionals (Figure 6A). Respondents’ most important requirements to work with AI were: adequate dental software/hardware, clear evidence of AI effectiveness, and evidence that dental experts have been involved in the development of the AI system (between 91–98% of participants expressed agreement) (Figure 6B). Visible support from or experience of academic parties, and positive expert opinions were important to respondents (81% and 78%, respectively, expressed agreement). In contrast, respondents were mostly neutral about or disagreed with requirements such as avoiding additional costs for users and exemption of liability for dental professionals (43% and 30%, respectively, expressed agreement).

**Fig. 7:**
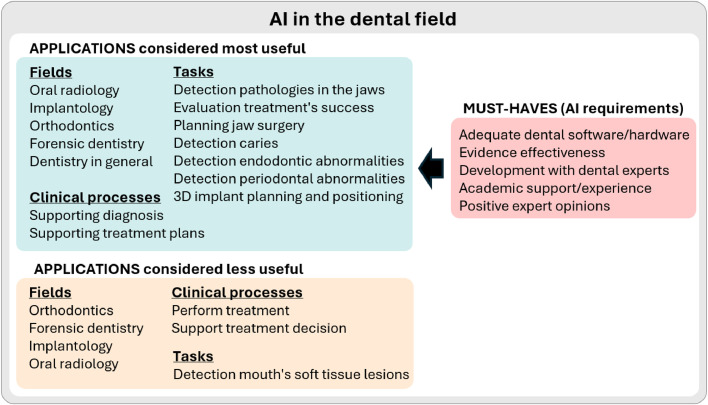
Summary of the perceived usefulness of applications of AI in the dental field, and desirable AI characteristics. For visualization purposes, an arbitrary cutoff of 70% was used.

Most respondents (n = 153, 92%) thought that AI education should be incorporated into training provided during dental conferences (67%), dental/medical school curricula (58%), and postgraduate dental programs (51%) (Figure 7).

## Discussion

Our results showed that dental students and professionals were positive about working with AI, regarding it as an opportunity to save time and improve care. This was consistent with our null hypothesis. They perceived AI as non-threatening and useful as a support tool across different dental fields and tasks. Figure 7 summarizes the most useful applications of AI (defined as those deemed useful by >70% of respondents), the less useful applications (deemed useful by ≤70% of respondents), and the desirable characteristics of these systems. The need for AI education was also highlighted.

Although there is, to our knowledge, no earlier report covering the acceptability of AI in the dental field in the Netherlands, a small but increasing number of reports from other countries have shown similar results: Yüzbaşıoğlu et al. in Turkey, Sur et al. in India, Pauwels and del Rey in Brazil, Jeong et al. in Korea, and Karan-Romero et al. and Salazar-Gamarra and Leon-Rios in Peru.^17^, ^18^, ^19^, ^20^, ^21^ For example, Jeong et al. found that Korean students and dentists believed that AI would become valuable in diagnosis and assisting treatment decisions.^20^ Similarly, dental professionals in India regarded AI as useful for (radiology-based) diagnosis.^18^ In Peru, dental students thought that AI could be used for treatment planning, quality control, and diagnostics.^21^ Moreover, our study found that students and dental professionals saw AI as a useful assistant to provide quality care, similar to other reports.^17^^,^^19^, ^20^, ^21^ The leading advantage of AI reported in our study is its time-saving potential. These perceptions are relevant in a context of global health workforce shortages, which also affect dental services in the Netherlands.^18^^,^ ^20^ Dutch dental professionals and students did not think that AI is better at making diagnoses or that it will make them redundant, a theme that is discussed widely in other contexts.^23^ These findings are in line with recent position papers from European dentistry organizations.^9^^,^^24^^,^^25^

Participants agreed that AI has limited capacity for empathy, similar to perceptions from South Korea.^20^ This contrasts with current views that AI systems could facilitate a better doctor-patient relationship, instead of limiting it,^26^ and the evidence that some AI systems are perceived as more empathic to patients than healthcare professionals.^27^ In the provision of care, attitudes such as empathy or compassion, are regarded as key to a good healthcare professional-patient relationship. Especially when an AI system directly interacts with patients, empathy is seen as a necessary feature, for example, for trust-building and treatment adherence.^28^ Although our results may suggest that empathy is a relevant feature of AI, it is necessary to research different types of AI applications and users’ (professionals and patients) requirements regarding empathy. For example, AI providing diagnostic support to dental professionals needs to be user-friendly and adapted to the workflow, but it may have very little or no “empathy requirements” if dental professionals deliver the results to their patients. This would place the AI as an assistant and the professional as the only responsible for providing empathic care.^9^

Accountability, responsibility and liability are challenging when AI systems are adopted into clinical workflows.^29^ Our results show that in the Netherlands, dental students and professionals accept at least some level of responsibility for the use of AI. At the same time, they expect AI manufacturers to share this responsibility. The developing European Union (EU) legal landscape, for example, the AI Act (Regulation (EU) 2024/1689) and the proposed AI Liability Directive, will hopefully help clarify questions regarding the liability of AI in healthcare, including the dental setting. However, at the moment here is a perception that EU regulation for AI in healthcare is too complex and unclear.^9^ Beyond the EU, other regions have no specific AI regulation, causing legal insecurity that may hinder the adoption of AI in healthcare. In both scenarios (with or without AI-specific regulation), there is a need to establish what dental professionals and patients expect and what is expected from them in terms of responsibility, liability, and the duty of care. We would like to emphasize that regulation alone is not expected to sufficiently cover all aspects deemed important by users.^9^^,^^25^^,^^30^^,^^31^ The same applies to other compliance requirements such as data protection and privacy (covered by the EU General Data Protection Regulation).

Our results support the view that the expected incursion of AI in the dental field demands the development of education programs at different levels.^17^^,^^19^^,^^20^^,^^32^ However, it is interesting that the majority of respondents favored training during conferences over the inclusion of AI education in dental/medical school curricula and postgraduate programs. We do not know the reasons behind this preference, and further research should be carried out before designing education or training programs. Nevertheless, we strongly recommend the structural incorporation of AI education into dental curricula^33^ and professional education plans, for example, through conferences, which are an important source of information for dental professionals.^9^^,^^24^ The design of these programs should carefully consider the most plausible AI clinical applications in dentistry, and prepare dental professionals to actively play a role in AI research, development, and implementation.

### Strengths and limitations

We did not find a validated questionnaire adequate for answering our research question, which stresses the need to work towards a more uniform methodology. Although the questionnaire was developed by an experienced and multidisciplinary team, it was based on previous literature, and it was tested on 3 individuals, no formal validation was carried out. For this reason, it is challenging to directly compare our results with other publications; however, we found insights that agree with previously reported data.

Our response rate was, in general, low. Because of privacy constraints, 2 recruitment mechanisms were used: via email and through an electronic newsletter. The expected response rate was 15–20%, which was only achieved for dentists, who were contacted directly by mail (20% response rate, n = 101). The response rate of dental students reached by mail was only around 6% (n = 57). Maxillofacial surgeons were invited via a newsletter, but they were underrepresented in our sample (n = 8). The interpretation of our results needs to consider that representativeness was limited by the response rates, especially in the case of maxillofacial surgeons. Since maxillofacial surgery is regarded as a field that could greatly benefit from AI,^13^ we strongly recommend future acceptability research focusing on this population. It was not possible to control for response bias. Because of statistical limitations, including the cross-sectional and survey-based nature of the study, no causal inferences could be drawn. Another limitation is that the attitudes towards AI evolve as technology and guidelines develop, and adoption in daily and professional life increases. This stresses the importance of carrying out follow-up research.

This study represents a valuable starting point to advance towards the responsible development and adoption of AI in the dental field.^34^ We strongly recommend more in-depth research that considers different AI applications in detail (e.g., general clinical processes and specific clinical tasks), and new AI technologies (e.g., those based on or assisted by generative AI). It would be interesting to determine the requirements to maximize benefits (advantages) and reduce risks (disadvantages) for the applications that showed higher acceptability. In the future, the acceptability of AI in the dental field among patients and other stakeholders, such as insurance companies and dental assistants, should be addressed. The reasons and motivations behind these perceptions should be studied in depth, using, for example, qualitative or mixed methods. This study is the first of its kind in the Dutch context and contributes to filling the literature gap about AI acceptability in the dental field. While our results indicate generally positive attitudes toward AI in dentistry, these findings should be interpreted in light of the study’s limitations, including possible selection bias, underrepresentation of certain subgroups, and the descriptive nature of our survey approach*.* Finally, we provided evidence to justify and inform the structural incorporation of AI education for dental professionals and students alike.

## Conclusion

AI-mediated innovation in dentistry is full of promises, but these benefits will not materialize without dental professionals’ acceptance of the technology. We showed that in the Netherlands, the acceptability of dental professionals and students is high when AI is developed to assist their work. Based on the areas of application regarded as most useful, this study can inform the prioritization of AI technologies for development, testing, piloting and implemention in clinical practice, as well as integration into educational programs. It serves as a starting point to inform the development of guidance and best practices, including clarifying the roles of dental professionals and patients and their corresponding responsibilities and liability. We make a call to the field, including students, professionals, professional organizations and academic institutions, to actively contribute to shaping the future of AI in dentistry.

## Cited regulations

Regulation (EU) 2024/1689 of the European Parliament and of the Council of 13 June 2024 laying down harmonised rules on artificial intelligence (AI Act), http://data.europa.eu/eli/reg/2024/1689/oj

Regulation (EU) 2016/679 of the European Parliament and of the Council of 27 April 2016 on the protection of natural persons with regard to the processing of personal data and on the free movement of such data, and repealing Directive 95/46/EC (General Data Protection Regulation (GDPR)), http://data.europa.eu/eli/reg/2016/679/oj

Proposal for a Directive of the European Parliament and of the Council on adapting non-contractual civil liability rules to artificial intelligence (proposed AI Liability Directive), https://eur-lex.europa.eu/legalcontent/EN/TXT/?uri=CELEX:52022PC0496

## Conflict of interest

The authors declare that they have no known competing financial interests or personal relationships that could have appeared to influence the work reported in this paper.

## Appendix Supplementary materials

Image, application 1

## Footnotes

Supplementary material associated with this article can be found in the online version at doi:10.1016/j.identj.2025.103933.
