# Supplementary material for: AI Acceptability in Dentistry: Insights from Dental Professionals and Students in the Netherlands: A Pilot Study
Source: Int Dent J. 2025 Oct 7;75(6):103933. doi: 10.1016/j.identj.2025.103933 (PMC12538124; doi:10.1016/j.identj.2025.103933)
Supplement: Supplementary file 1 [file mmc1.docx]

**Supplemental files.**

**AI Acceptability in Dentistry: Insights from dental professionals and students in the Netherlands, a pilot study**

**Supplemental file 1****. Questionnaire (English translation).**

The Dutch version is available upon request.

**1 GENERAL**

1.1 Age in years

_________

1.2 Gender

- Man (1)
- Woman (2)
- I'd rather not say (3)

1.3 Profession

- Student (1)
- Dentist (2)
- Orthodontist (3)
- Maxillofacial surgeon (4)

Display This Question: If 1.3 = 1

1.4 Dentistry/Medicine

- Dentistry (1)
- Medicine (2)

Display This Question: If 1.4 = 1

1.5 Year of study

- Bachelor 1 (1)
- Bachelor 2 (2)
- Bachelor 3 (3)
- Master 1 (4)
- Master 2 (5)
- Master 3 (6)

Display This Question: If 1.4 = 2

1.5 Year of study

- Bachelor 1 (1)
- Bachelor 2 (2)
- Bachelor 3 (3)
- Master 1 (4)
- Master 2 (5)
- Master 3 (6)

Display This Question: If 1.3 = 2

1.4 General practitioner/Specialized dentist
If you work as both a general and a specialized dentist, choose the option in which you are most active.

- General practitioner (1)
- Specialized dentist (2)

Display This Question: If 1.4 = 1

1.5 Years of experience

_______________

Display This Question: If 1.4 = 2

1.5 Please specify your dental specialty

____________________

Display This Question: If 1.4 = 2

1.6 Years of experience

_______________

Display This Question: If 1.3 = 3

1.4 Orthodontist in training/practicing orthodontist

- Orthodontist in training (1)
- Practicing Orthodontist (2)

Display This Question: If 1.4 = 1

1.5 Year of training

- 1 (1)
- 2 (2)
- 3 (3)
- 4 (4)

Display This Question: If 1.4 = 2

1.5 Years of experience

________________

Display This Question: If 1.3 = 4

1.4 Maxillofacial surgeon in training/practicing maxillofacial surgeon

- Maxillofacial surgeon in training (1)
- Practicing maxillofacial surgeon (2)

Display This Question: If 1.4 = 1

1.5 Year of training

- 1 (1)
- 2 (2)
- 3 (3)
- 4 (4)

Display This Question: If 1.4 = 2

1.5 Years of experience (after completing the training)

________________________________________

**2 GENERAL KNOWLEDGE ABOUT THE CURRENT USE OF ARTIFICIAL INTELLIGENCE (AI) IN EVERYDAY LIFE**

2.1 AI already has many applications in our daily lives. Some examples include Amazon Alexa or Google Assistant voice and text recognition, spam filters for mail, and advertisement recommendation algorithms that are active when you browse the web. Are you aware of the role that AI already plays in your daily life, as illustrated by the examples above?

- Completely unaware (1)
- Moderately unaware (2)
- Neutral (3)
- Moderately aware (4)
- Completely aware (5)

**3 POSSIBLE APPLICATIONS OF AI IN DENTISTRY**

Display This Question: If 1.3 = 1, = 2, or = 3

3.1 AI plays an important role not only in everyday life, but also in the medical world. The following questions refer to the possible role of AI in dentistry. For each example, indicate the extent to which you agree with it.

AI can have useful applications in the following dental fields:

|  | Strongly disagree (1) | Moderately disagree (2) | Neutral (3) | Moderately agree (4) | Strongly agree (5) |
| --- | --- | --- | --- | --- | --- |
| Dentistry in general (1) |  |  |  |  |  |
| Preventive dentistry (2) |  |  |  |  |  |
| Restorative dentistry (3) |  |  |  |  |  |
| Oral Radiology (4) |  |  |  |  |  |
| Implantology (5) |  |  |  |  |  |
| Orthodontics (6) |  |  |  |  |  |
| Periodontology (7) |  |  |  |  |  |
| Endodontology (8) |  |  |  |  |  |
| Forensic Dentistry (9) |  |  |  |  |  |

Display This Question: If 1.3 = 4

3.1 AI plays an important role not only in everyday life, but also in the medical world. The following questions refer to the possible role of AI in dentistry. For each example, indicate the extent to which you agree with it.

AI can have useful applications in the following dental fields:

|  | Strongly disagree (1) | Moderately disagree (2) | Neutral (3) | Moderately agree (4) | Strongly  agree (5) |
| --- | --- | --- | --- | --- | --- |
| Dentistry in general (1) |  |  |  |  |  |
| Oral Radiology (2) |  |  |  |  |  |
| Implantology (3) |  |  |  |  |  |
| Orthodontics (4) |  |  |  |  |  |
| Periodontology (5) |  |  |  |  |  |
| Endodontology (6) |  |  |  |  |  |
| Forensic Dentistry (7) |  |  |  |  |  |

3.2 AI could have multiple applications within these different fields. Several examples are described below. Indicate the extent to which you agree with the following statements.

AI can support with:

|  | Strongly disagree (1) | Moderately disagree (2) | Neutral (3) | Moderately agree (4) | Strongly  agree (5) |
| --- | --- | --- | --- | --- | --- |
| Making a diagnosis (1) |  |  |  |  |  |
| Treatment planning (2) |  |  |  |  |  |
| Making treatment decisions (3) |  |  |  |  |  |
| Performing a treatment (4) |  |  |  |  |  |

3.3 The following are specific examples of possible tasks for AI, related to specific areas within dentistry. Indicate the extent to which you agree with each example.

AI could support:

|  | Strongly disagree (1) | Moderately disagree (2) | Neutral (3) | Moderately agree (4) | Strongly agree (5) |
| --- | --- | --- | --- | --- | --- |
| In the detection of caries (1) |  |  |  |  |  |
| In the detection of periodontal diseases (2) |  |  |  |  |  |
| In the detection of endodontic problems such as periapical lesions (3) |  |  |  |  |  |
| In the detection of soft tissue lesions of the mouth (4) |  |  |  |  |  |
| In the detection of pathology in the jaws, such as osteomyelitis (5) |  |  |  |  |  |
| In 3D implant positioning and planning (6) |  |  |  |  |  |
| When planning oral surgeries such as osteotomies (7) |  |  |  |  |  |
| As a “quality control tool” to evaluate the success of treatments, for example, in assessing the healing of periapical lesions after endodontic treatments or the quality of a canal filling (8) |  |  |  |  |  |
| In preventive dentistry, e.g., in applications in toothbrushes that can detect plaque (9) |  |  |  |  |  |

**4 Advantages and disadvantages for users**

4.1 The application of AI in daily practice could have advantages and/or disadvantages. Below you will find statements about AI. For each of the examples below, please indicate the extent to which you agree.

|  | Strongly disagree (1) | Moderately disagree (2) | Neutral (3) | Moderately agree (4) | Strongly agree (5) |
| --- | --- | --- | --- | --- | --- |
| AI can improve patient care (1) |  |  |  |  |  |
| AI can help save costs (2) |  |  |  |  |  |
| AI can save time (3) |  |  |  |  |  |
| AI can reduce medical/dental errors (e.g., missing pathology) (4) |  |  |  |  |  |
| AI can act as a second opinion (5) |  |  |  |  |  |
| AI has a poorer capacity for empathy and to consider the emotional well-being of the patient (6) |  |  |  |  |  |
| The diagnostic ability of AI is superior to that of a dental professional (7) |  |  |  |  |  |
| AI can make wrong diagnoses (8) |  |  |  |  |  |
| AI can lead to overtreatment (9) |  |  |  |  |  |

4.2 Who do you think should be held liable for medical and/or legal problems caused by AI?

|  | Strongly disagree (1) | Moderately disagree (2) | Neutral (3) | Moderately agree (4) | Strongly agree (5) |
| --- | --- | --- | --- | --- | --- |
| The dental professional (1) |  |  |  |  |  |
| The company that developed the AI (2) |  |  |  |  |  |
| The patient who consented to follow the AI's input (3) |  |  |  |  |  |

**5 Outcome Expectations**

5.1 Should AI be part of education at any of these levels? (multiple answers are possible)

- Dental/medical school (1)
- Postgraduate programs (2)
- Dental conferences (3)
- None (4)

5.2 AI will make the dental professional redundant.

- Strongly disagree (1)
- Moderately disagree (2)
- Neutral (3)
- Moderately agree (4)
- Strongly agree (5)

5.3 Would you be willing to work with AI technology as a dental professional?

- Yes (1)
- No (2)
- Not sure (3)

Display This Question: If 5.3 = 1, 5.3 = 3

5.4 What would be important for you to work with AI technology?

|  | Strongly disagree (1) | Moderately disagree (2) | Neutral (3) | Moderately agree (4) | Strongly agree (5) |
| --- | --- | --- | --- | --- | --- |
| Clear evidence of AI’s effectiveness (1) |  |  |  |  |  |
| Visible endorsement and experience from professors/universities (2) |  |  |  |  |  |
| Positive expert opinions (3) |  |  |  |  |  |
| No extra costs for the user (4) |  |  |  |  |  |
| No liability (5) |  |  |  |  |  |
| Well-incorporated dental software/hardware (6) |  |  |  |  |  |
| Involvement of dental experts in the development of AI (systems) (7) |  |  |  |  |  |
| Something else (please specify) (8): |  | | | | |

Display This Question: If 5.3 = 2

5.4 What would be needed for you to be willing to work with AI technology?

|  | Strongly disagree (1) | Moderately disagree (2) | Neutral (3) | Moderately agree (4) | Strongly agree (5) |
| --- | --- | --- | --- | --- | --- |
| More evidence of AI’s effectiveness (1) |  |  |  |  |  |
| More visible endorsement and experience from  professors/universities (2) |  |  |  |  |  |
| Positive expert opinions (3) |  |  |  |  |  |
| No extra costs for the user (4) |  |  |  |  |  |
| No liability (5) |  |  |  |  |  |
| Well-incorporated dental software/hardware (6) |  |  |  |  |  |
| Involvement of dental experts in the development of AI (systems) (7) |  |  |  |  |  |
| Something else (please specify) (8): |  | | | | |

5.5 If you were a patient, would you want your oral healthcare professional to use AI technology?

- Strongly disagree (1)
- Moderately disagree (2)
- Neutral (3)
- Moderately agree (4)
- Strongly agree (5)
